# Perioperative Point-of-Care Ultrasound in Children

**DOI:** 10.3390/children7110213

**Published:** 2020-11-06

**Authors:** Karen Boretsky

**Affiliations:** Department of Anesthesiology, Critical Care and Pain Medicine, Boston Children’s Hospital, Harvard Medical School, Boston, MA 02115, USA; karen.boretsky@childrens.harvard.edu

**Keywords:** pediatric, anesthesia, point-of-care, bedside ultrasound, surgery, perioperative, diagnostic imaging

## Abstract

Anesthesiologists and other acute care physicians perform and interpret portable ultrasonography—point-of-care ultrasound (POCUS)—at a child’s bedside, in the perioperative period. In addition to the established procedural use for central line and nerve block placement, POCUS is being used to guide critical clinical decisions in real-time. Diagnostic point-of-care applications most relevant to the pediatric anesthesiologist include lung ultrasound for assessment of endotracheal tube size and position, pneumothorax, pleural effusion, pneumonia, and atelectasis; cardiac ultrasound for global cardiac function and hydration status, and gastric ultrasound for aspiration risk stratification. This article reviews and discusses select literature regarding the use of various applications of point-of-care ultrasonography in the perioperative period.

## 1. Introduction

Bedside ultrasound-imaging, point-of-care ultrasound (POCUS), was first introduced into the operating rooms in 1984 as a procedural adjunct to facilitate placement of central intravenous access [1]. While originally resisted by the anesthesia community, the use of ultrasonography for central line placement has decreased infection rates, decreased the incidence of complications (pneumothorax and bleeding), decreased the time for placement, and increased first-attempt and overall success rate [2,3]. The Agency for Healthcare Research and Quality has named ultrasound use for central line placement as one of the 12 most highly rated safety practices [4]. Ultrasonography is now a standard of care for central line insertion. Similarly, the application of ultrasound to localize nerves when performing pediatric regional anesthesia has increased success rates, decreased block performance times, and lowered the incidence of complications, including seizures and cardiac arrest [5,6]. The most common regional anesthetic performed in children is caudal blockade and success rates are increased to 95–100% with ultrasound guidance compared to 79–80% with landmark technique [7,8,9]. In infants, ultrasound can be used to verify epidural catheter placement through the acoustic window provided by the incomplete ossification of the infant spine [10,11,12]. This spares additional exposure to radiation otherwise needed for confirmatory epidurograms. New and safer regional anesthetic procedures are possible due to the increased imaging capability provided by ultrasonography [13,14,15].

The greatest impact on patient care may, however, be the emerging use of POCUS as a diagnostic tool in response to deteriorating hemodynamic or pulmonary status. POCUS can answer specific binary (yes or no) questions in real-time to guide critical clinical decisions regarding endotracheal tube size and position, lung ventilation, and lung pathology, global cardiac function and hydration status, and to stratify aspiration risk with gastric imaging [16,17,18,19]. POCUS is distinct from radiology-performed ultrasound, in that the clinician integrates the results with the clinical history and physical examination in real-time at the bedside. The purpose of this article is to describe specific POCUS applications performed on children in the perioperative period and discuss benefits and limitations compared with conventional practice.

## 2. Lungs and Airway

Lung ultrasound has become a fundamental application for POCUS in many specialties in both pediatric and adult patient populations [20]. POCUS studies in pediatric operative populations have focused on the identification of proper endotracheal tube position and diagnosis of pneumonia, atelectasis, pneumothorax, and pleural effusion.

**Figure 1:**
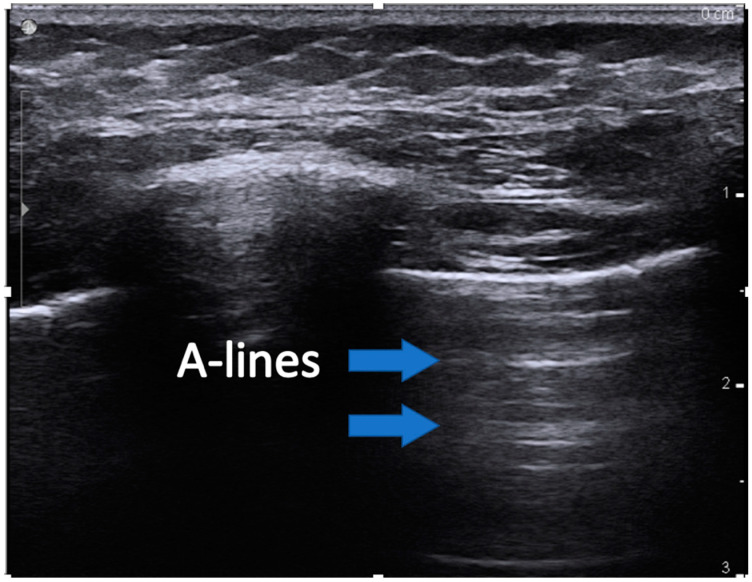
Image showing an example of A-lines. The blue arrows indicate the reverberation artifact lines at recurring regular intervals.

**Figure 2:**
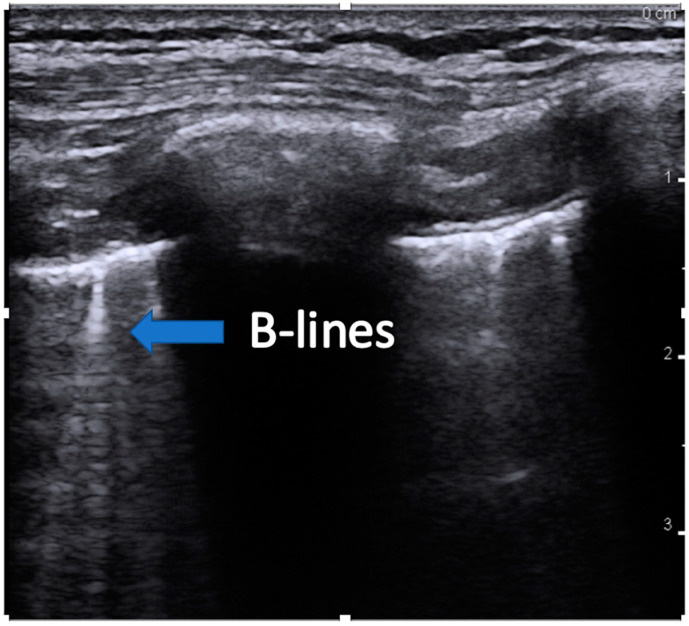
Image showing an example of B-lines. The blue arrow indicates the long hyperechoic lines that continue the full depth of the image, indicating replacement of air-filled alveoli.

Common ultrasound terms and findings are listed [16]:

(1) Lung sliding is a visualization of pleural movement between adjacent ribs. With respiration, lung movement causes the parietal and visceral pleura to slide against each other, creating the appearance of a shimmering linear structure referred to as “lung-sliding”.
(2) Lung pulse refers to the pleural movement concurrent with cardiac contraction. Pulsations of blood flow result in small changes in lung volume, creating pleural movement.
(3) A-lines are horizontal artifacts produced by air in the chest cavity. Air reflects ultrasound waves and prevents the visibility of structures beneath. Some of the reflected ultrasound waves, however, bounce back and forth between the muscle, and fascia of the chest wall and return to the transducer, generating reverberation artifact called a-lines (Figure 1).
(4) B-lines are created when air is replaced in the alveoli by fluid, septal thickening, or other interstitial lung disease and which transmit the sound waves. B-lines originate at the pleural line and are long, vertical hyperechoic lines traversing the entire image depth (Figure 2). Up to two B-lines per rib space are normal, especially in dependent areas of the lung.

Sudden and/or gradual decreases in oxygen saturation accompanied by deteriorating vital signs is not an uncommon scenario during pediatric surgery, especially in mechanically ventilated infants and children with severe lung disease or those patients undergoing bronchoscopy or thoraco-abdominal surgery. The differential diagnosis includes (but is not limited to) endobronchial intubation and pneumothorax, which can both be identified by a POCUS exam [17,21,22]. A pneumothorax can be rapidly ruled out with POCUS by the presence of lung sliding, lung pulse, or B-lines. Endobronchial intubation and endobronchial tube migration are common in children, due to their short tracheal length. Patient positioning, repositioning, and insufflation are associated with endotracheal tube (ETT) migration and location [23,24]. Lung sliding will be absent when respiration is absent, which most commonly occurs during endobronchial intubation of the contralateral side, ipsilateral bronchial obstruction (foreign body, clot, mucous), or esophageal intubation. While auscultation is currently the gold standard for confirmation of ETT position, it fails to identify as many as 38% of endobronchial intubations in adult elective intubations and 5% of pediatric intubations [25]. Recent studies demonstrate the ability to use point-of-care ultrasound to verify bilateral ventilation by examining lung sliding and a lung pulse [26,27]. Ultrasound imaging correctly identified endobronchial intubation in 95–100% of adults and children compared to 62% using auscultation [21,22]. A lack of pleural sliding can, however, also indicate bronchial obstruction or pneumothorax. Lung ultrasound can also be used to diagnose atelectasis, hemothorax, and to determine diaphragmatic movement after surgery. If a child presents for emergency surgery and pneumonia is suspected, lung POCUS is highly accurate in diagnosing pneumonia in children when compared with chest x-ray CXR [28,29,30]. Regarding POCUS, pneumonia may be seen as subpleural consolidations or “hepatization” of the lung [30,31].

**Figure 3:**
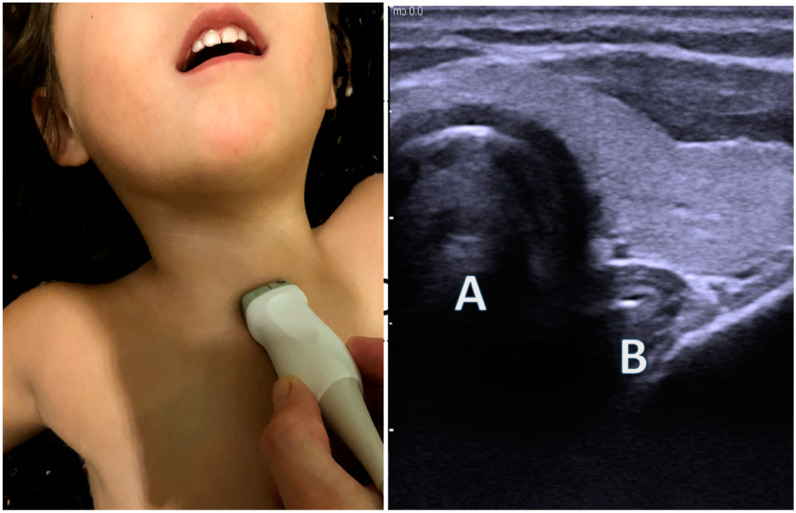
Image showing the placement of a high-frequency linear transducer just above the sternal notch with resultant ultrasound images. A = trachea and B = esophagus.

Inadvertent esophageal intubation during attempted endotracheal intubation occurs in up to 21% of infants with a 4%incidence of hypotension and the initiation of chest compressions in 3% of esophageal intubated patients [32]. This makes the placement of ETT in infants a potentially high-risk procedure with good patient outcomes dependent on rapid verification of ETT position. Ultrasound imaging over the trachea directly above the sternal notch images both the trachea and the esophagus with the esophagus located laterally (Figure 3). During intubation, observation of an empty esophagus and widening subglottis indicates successful tracheal intubation while the paratracheal appearance of the ETT in the esophagus, “double trachea sign”, [33] indicates esophageal intubation. Adult and pediatric literature show sensitivity and specificity of 98.5–100% and 75–100%, respectively, for diagnosis of esophageal intubation [21,34,35].

## 3. Focused Cardiac Ultrasound (FOCUS)

Transthoracic POCUS of the heart, or focused cardiac ultrasound (FOCUS), is used in hemodynamically unstable children to narrow the differential diagnosis and guide care [17,36,37,38]. FOCUS is used to diagnose pericardial effusions, assess for asystole, and assess global function and contractility [39,40,41,42,43]. It is important to note that FOCUS is not meant to identify complex congenital heart disease or replace a comprehensive echocardiogram. The interpretation of echocardiography in children with structural congenital heart disease (CHD) is difficult, and FOCUS in children with CHD is limited to identification of acute conditions, such as a pericardial effusion or air embolus, and to assess overall myocardial function [17,37,39].

The most common indications for FOCUS in children are to diagnose etiologies for hypotension, tachycardia, and reversible causes of cardiac arrest [17,39]. It can help distinguish between the causes of shock. While pediatric perioperative data is still scarce, FOCUS shortens the time to make a diagnosis and institute appropriate therapy in pediatric emergency departments and intensive care unit (ICU) [40,41,42,43,44,45,46] and improves outcomes [45]. Serial FOCUS exams can be used to monitor intervention and confirm the resolution of pathology in real-time [44,47,48].

A linear phased array transducer oscillating at 1–5 MHz with a 2–3 cm square footprint produces satisfactory image quality and is the FOCUS standard in children of all sizes and eliminates the need for multiple transducers [36]. A single go-to transducer eliminates the time and distraction of selecting and locating transducers. When a phased array transducer is not available, a convex transducer with a small footprint provides good quality images in infants and smaller children in the subcostal and parasternal windows [36].

The standard windows and views are described in published overviews of pediatric FOCUS [36,42].

**Figure 4:**
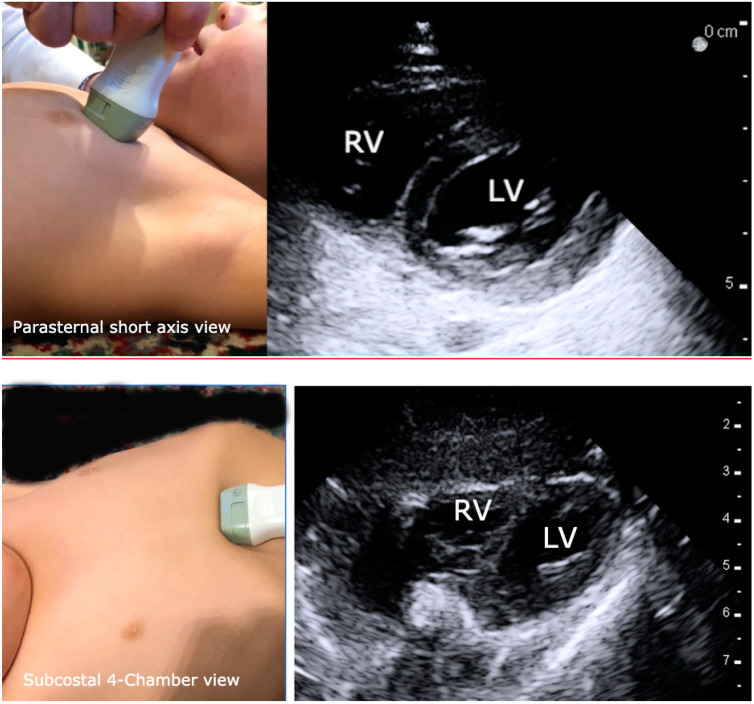
Images that illustrate proper transducer placement and resultant normal images for the parasternal short axis and subcostal 4-chamber views of the heart. RV = right ventricle and LV = left ventricle.

The subcostal 4-chamber (S4CH) view and the parasternal short axis (PSAX) are easiest to learn and ideal for assessing left ventricle (LV) size and systolic function, movement of the intraventricular septum, and to evaluate for pericardial effusion or emboli (Figure 4). The parasternal short axis PSAX window is generally the most accessible in children who are draped for surgery (Figure 4). The parasternal long-axis (PLAX) view is best for gross valvular assessment. The S4CH view is the recommended imaging window during cardiopulmonary resuscitation CPR [49,50,51] because it is the least disruptive of chest compressions. When possible, several views should be obtained to confirm the interpretation.

There are some differences when comparing imaging of children to adults. The closer proximity of the heart to the chest wall permits higher frequency imaging, and thus, more detailed imaging. In the operating room, FOCUS is mostly performed on anesthetized children. Challenges arise from smaller target structures, faster heart rates, probe to patient size mismatch, limited access to small patients under surgical drapes, and potentially uncooperative awake patients.

Quantitative calculations are uncommonly used in pediatric FOCUS, and qualitative assessment is more important. Studies of FOCUS on children have demonstrated a good correlation between visually estimated (qualitative) and measured (quantitative) cardiac ejection fraction [52,53].

Interpretation must always consider the patient history and related pathologies [36,37,38]. Compared to adults, infants, and smaller children, have a higher incidence of local anesthetic systemic toxicity, hemodynamically significant air emboli, and cardiac arrest secondary to respiratory insufficiency [33,54,55]. Adverse intraoperative respiratory events are common in children, with up to 40% of intraoperative cardiac arrests resulting from a respiratory etiology [55]. FOCUS can differentiate between a primary respiratory problem with secondary cardiac arrest and cardiac etiologies of cardiac arrest [56,57].

FOCUS can determine cardiac activity in the presence or absence of a pulse [58]. This provides critical information in children where pulse palpation is unreliable [59,60].

In the setting of cardiac arrest, FOCUS is used to obtain information about potentially reversible etiologies. This can be useful to rapidly direct appropriate care. Reversible causes of cardiac arrest, including pericardial tamponade, hypovolemia, and pulmonary, and air embolus, can all be confirmed with FOCUS [49,50,51,61,62]. The time allowed for an ultrasound exam during CPR is strictly limited to the 10-s pause performed every minutes per Pediatric Advanced Life Support (PALS) protocol. A rapid transition to extra corporeal membrane oxygenation (ECMO) for failed cardiopulmonary resuscitation, (E-CPR,) is especially important in children with ventricular asystole [62] with evidence of improved survival in pediatric patients with in-hospital arrest requiring >10 min of standard CPR. There is also evidence that prolonged conventional CPR with ongoing use of epinephrine elevates systemic vascular resistance and may limit ECMO pump flows when implementing E-CPR [63]. FOCUS may decrease epinephrine use and/or time to E-CPR.

## 4. Gastric

Pulmonary aspiration of gastric contents occurs in approximately 0.4–0.1% of anesthetics in children and can lead to hypoxia, pneumonia, and prolonged mechanical ventilation [55,64,65,66]. The mainstay of aspiration prevention has been adherence to standardized nil-per-os (NPO) guidelines, but interpatient variability in gastric emptying, co-morbidities, and ambiguous NPO status can present uncertainty of degree of risk. The clinical value of gastric ultrasound to stratify risk for aspiration is documented in a few case reports and small series [67,68].

Bedside ultrasound-imaging using a standard examination and measurement of the antral cross-sectional area is validated to predict gastric volume and content in both adult and pediatric patients [69,70,71,72,73]. Patients are classified according to the character and volume of gastric contents using antral grades 0–2. Grade 0 is an empty antrum in both supine and right lateral decubitus position (RLD) and correlates with negligible gastric volume. A grade 1 antrum is defined as clear fluid (anechoic content) seen in the RLD only, but not in the supine position. Both grade 0 and grade 1 antrums are common in fasting children (>95% of cases) and correlate with low volumes of gastric fluid consistent with normal baseline secretions (<1.5 mL/kg). Antral grade 2 is defined as visible clear fluid in both supine and RLD positions. A grade 2 antrum is uncommon in fasting children (<5% of cases) and correlates with a gastric volume >1.5 mL/kg. When a grade 2 antrum is identified, or any amount of solid (heterogeneous/particulate) or thick fluid content (hyperechoic) is observed, there is a concern for increased risk for aspiration with surgery delayed or the anesthetic modified as indicated.

## 5. Conclusions

POCUS has the potential to improve care for perioperative pediatric patients. Although there is little research for POCUS applications specific to this hospital setting, there are well-established applications in adult and other pediatric subspecialties that may be applied in the perioperative pediatric setting. Rigorous training and credentialing processes should be implemented to make POCUS available to all children under the care of anesthesiologists and other acute care practitioners throughout the perioperative period.
